# Remarks on Insanity; Founded on the Practice of John Mayo, M. D. Fellow of the College of Physicians, and Tending to Illustrate the Physical Symptoms and Treatment of the Disease

**Published:** 1818-01

**Authors:** 


### PART II.

### COMPREHENSIVE ANALYTICAL REVIEW OF MEDICAL LITERATURE.

"Tros, tyriusve, nobis nullo discrimine agetur."

*Remarks on Insanity; founded on the Practice of* JOHN MAYO, M. D. *Fellow of the College of Physicians, and tending to illustrate the Physical Symptoms and Treatment of the Disease.* By THOMAS MAYO, B. M. Fellow of Oriel College, Oxford. Octavo, pp. 90. London, 1817.

THE exposition before the Committee of Inquiry, of the horrible scenes practiced in some of the Lunatic Asyums, together with the Bill now pending for the better regulation of Mad Houses, have lately directed the attention of physicians to the nature and causes of that most formidable and afflictive disease Insanity; and, whatever may be the motives of the writers on this important subject, it is to be hoped that something useful may be collected from the whole, and that the time is approaching, when the treatment of this malady will be entirely rescued from the hands of the empiric, or self-created philosopher, and entrusted to the care of the intelligent and careful physician of established reputation, under certain retrictions, which woeful experience of the unnecessary and shameful cruelties inflicted on the unfortunate and helpless manaiac, has taught us to be absolutely required. Yet, though the urgent necessity of legislative interference is obvious to the meanest capacity, we would wish that the mode of this interference should be most maturely deliberated on, so that the inconscious being, for whose benefit it is intended, may have every protection; and that the physical and moral treatment may have as little interruption as possible.

Dr. Mayo, in the elegant and classical little book before us, professes that the only objects he aims at, are the vindication of the rights of his profession over insanity, and the elucidation of its medical treatment. Our opinion perfectly coincides with the Doctor's, that this disease is peculiarly the province of the physician, and until it is entirely under his management, we cannot look forward with confidence to a correct history of the nature, causes, symptoms, or treatment of the various species of mental alienation, which we have invariably found upon minute investigation, connected or combined, more or less, with hepatic or other organic affections. But, in order that a precise view of the mental aberrations may be exhibited, we strenuously recommend that the most minute attention should be paid to the precursory symptoms, and to the more delicate shades of the disease. It is, indeed, surprizing, upon a careful investigation of the primary affection, how insidiously it makes its approaches, and what variety of appearances it assumes! In many cases two, three, six, ten, or twelve months before it has been identified as insanity, the near relatives and friends of the patient may discover that his stomach and bowels are disordered; that his spirits droop; he becomes heavy, absent, and abstracted; seeking solitude, and forsaking all his former enjoyments; his temper becomes irascible; and nothing gives him pleasure or gratification; he fancies all around him are his enemies; he speaks quick, and hurriedly; frequently faltering, and delivering himself in half sentences; he is petulant, fretful, jealous and uneasy! All this is attributed to something that has soured his temper, and passes on from day to day with nothing but an exclamation of, What can be the matter! How much he is altered! He is hardly like the same person! How cross he has become! Never dreaming that disease at this time is making most rapid advances, until at last, a deed of desperation flashes conviction on the unobservant friends; and by the combination of little circumstances, which at the time were scarely noticed, a chain of connection is traced, which recalls the most painful recollection, that had timely measures been pursued, the fatal catastrophe might have been prevented. We have sometimes flattered ourselves, that we have prevented a fit of insanity by prompt and decisive measures; and the patient now before us, who has been twice in a state which rendered confinement indispensable, is an illustration of what we have advanced.

The husband of this woman, aged 40, being aware of the mode of its attack, and fearing that his children might again be deprived of a most affectionate mother, requested us to visit her about ten days ago. We found her complaining of great lowness and depression of spirits; slight pain and uneasiness at the præcordia, particularly on pressure, and calling her attention to that point; some slight uneasiness in the head, and wandering of the imagination: The bowels were constipated; the eyes heavy and dull; and the countenance exhibited that peculiar appearance, which those accustomed to the disease readily recognize. There was a sense of oppression and fulness; and the belly was swelled and flatulent; for several preceding nights she had been restless, and harassed by the most terrific and horrible dreams. The husband declared that these symptoms had always preceded her former attacks of insanity. A copious and sudden abstraction of blood, aided by a brisk cathartic, completely relieved her; and as her bowels are still inclined to costiveness, and her stools discoloured and fœtid, she continues the cathartic at intervals; but her understanding is clear, and she is convalescent.

Dr. Mayo commences by informing us, that it is his intention to comprehend those forms of madness which are viewed under the heads "Maniac and Melancholic," by Doctors Pinel, Esquinol, and Dubnisson; and, in a note, he observes, that his reason for resorting to France for a history of insanity is, that he is bound to seek information wherever it is given with the greatest accuracy, and to the greatest extent.

"Let it be remembered, that the French revolution has given the physicians of that country an advantage, to which we have nothing analogous, in supplying an immense mass of simultaneous cases."—Note, page 1, 2.

Now we have often heard of the immense increase of insanity in that country since the commencement of the revolution; but on a minute inquiry in Paris and other cities, we have reason to conclude, that this statement is founded on error and exaggeration. During the first years of that eventful period, many were thrown into a state of insanity by the sudden changes from affluence and splendour to wretchedness and poverty; others, by the loss of their families or friends; some few by disappointed ambition; and others again, by the rapid success they obtained. At such a time, when the passions of man are roused to the highest pitch, we may expect that reason will frequently forsake its seat; yet, during the despotic reign of Buonaparte, the number has not increased beyond what might be expected from an increasing population; but, the proportion of young to old men has been much greater. This is attributed by the most intelligent French physicians, to the rigour of the Conscription. Upon an impartial view of the subject, it appears to us, that the fluctuation of property, arising from our commercial speculations, has had as great an effect in producing insanity in this country, as the changes of government and property in France. Besides, it is notorious that the decrease of religious maniacs, is equal to the increase produced by political opinions.

The first chapter of the volume, now under our consideration, is occupied in reviewing the opinions of the most celebrated French and English authors on Insanity, and in endeavouring to draw the attention to the physical pathology of the disease: the author's views will be comprehended from the extracts we shall lay before our readers, and will exhibit a specimen of the style and language in which the work is written.

"To regard insanity as an object of attention with reference to its mental phænomena, while the disease is quiescent; and to repress it by coercion and medical measures while it is raging, is the first plausible suggestion which would occur to the physician.

"The disease, under this treatment, appeared to my father uncertain of obtaining a present cure; the patient was very liable to relapse. He looked more attentively at physical symptoms; he saw the insidious ravages of the disease in derangement of the general bodily functions, even while it was apparently quiescent. He saw slight but daily exacerbations indicated by increased action of the carotids and flushing of the face, and heightened from time to time into forms of violent febrile excitation. At length, when the slow process should have achieved its purpose, when disorganization of the part might be supposed to have taken place, he saw the functions of that part, namely, the mental powers, sink into such total ruin, as morbid anatomy teaches us to associate with disorganized structure." page 11—12.

Our author then continues to illustrate this view of the subject by a reference to Mr. J. Haslam's observations on madness and melancholy, and clearly demonstrates from the phænomenon of the thickening, or opacity of the tunica arachnöides, together with other phænomena from the same source, that in the most advanced stages of the disease, even where we would be inclined to argue, *à priori*, against this indication, there is a continued process of increased action of a chronic nature.

We perfectly coincide with the Doctor in the following remarks on the pathology and treatment of the disease.

"As attended by increased activity in the circulation of the brain, mania appears to require depletory treatment. In as far as this increase of vascular activity is prolonged and uninterrupted, in so far the discipline, with which it is combated, must possess these qualities. In such discipline the means must apparently be sought of combating disease, which lurks in an insidious form, even after its active effervescence may have been subdued. If in phrenitis active symptoms yield, before some fatal effusion has taken place, the patient is cured.—But, though the paroxysm of mania be subdued, it leaves a state of mitigated excitement behind, which, unless followed to its close by depletion, will revive the disease.

"The slow process of morbid action shews itself not merely by symptoms directly connected with the disease, but also by its effect on the general habit of the body. The stomach, the liver, the spleen, the intestinal canal, the heart, even respiration, labours under this influence. It is not until these sympathetic disorders are quieted, that a satisfactory cure can be asserted. Certain points are observable in the progression of the disease, which have peculiarly a reference to its treatment. They are, indeed, established by nature; and exist, in the more or the less, where the disease is either absolutely neglected, or differently treated; but they are strongly marked where that plan of treatment is pursued which we recommend." page 19—20.

In sketching the physical symptoms in the incipient stage, our author proceeds to recommend bleeding and cupping, and offers the following judicious observations on the sub-acute state of inflammation.

"In such a state of disease, venæsection or cupping will often produce evident temporary excitement, perhaps amounting to a paroxysm. Often, during the remittent stage, bleeding has evidently occasioned head-ache, where head-ache has been the only phænomenon wanting to complete a set of symptoms usually co-existent. The history of apoplexy points out with sufficient evidence, that if there is a state of overactive circulation which produces head-ache, this state, heightened to a more intense degree, will produce a heavy, stupid feeling, rather than pain. Thus the process, which lessens vascular action, may occasion head-ache." page 24.

We cannot abbreviate the enumeration of the means of cure.

"They consist of occasional cupping or bleeding; of the use of issues or setons; of continued purgation; of nauseating medicines; finally, of the class of sudorific and refrigerant medicines. To this last mentioned class of remedies we attach, however, very secondary importance." page 26.

The Doctor proceeds to point out some of the effects of purgatives in producing flatulence. In the description of the approach of a paroxysm, we can see nothing new or particularly worthy of observation; the state of the patient is alone capable of directing the physician how far depletion may be carried. After some excellent remarks on the remittent and continuous stages of the disease, and on the means of wielding the instruments recommended, the author advances to the symptomatic or simultaneous derangement of other viscera besides the brain, which occur in insanity, and exhibits the influence exerted by such derangement in the modification of the disease; he ultimately describes some of the irregularities which frequently occur in mania.

In considering the objections to the depletory system, our author states the opinion of some of the French physicians, that depletion pushed far, and long continued, leads to a passive form of the disease very difficult of cure, if not incurable. We have seen the French mode of depletion, and do not wonder at the result. We would contrast their method with that of Dr. Armstrong, contained in his invaluable work on Typhus, which we cannot too often recommend to the perusal of our readers.

Our limits will not admit of more copious extracts from this interesting little volume, which confirms our preconceived opinion, that more attention should be paid to the physical as well as the mental symptoms; but, before we take leave, we cannot avoid observing that it appears, the Doctor, like many more of our countrymen, has been searching abroad for information, while he has passed over much which he might have acquired at home; and it is not without some feelings of surprize, that we have perceived no mention of the inestimable work of Dr. Armstrong, to which we have already alluded; more particularly as the conclusions of Dr. Mayo are similar, in many points, to those maintained in that work.

We shall trespass no further on the patience of our readers than in extracting the concluding passages, with which we fully coincide.

"One principal object of the remarks which are now concluded, has been to arrest the encroachments of metaphysical speculation on the pathology of madness. As I have laboured to separate and define its domain on this side, so I would wish to destroy bounds and enclosures unjustly assigned it on another. If it be inexpedient that this disease should become a province of psychology, it is almost equally inexpedient that it should constitute a study distinct and separate from that of general disease.

There is no complaint which has contracted more extensive alliances than this one. Its citadel is indeed the brain; but that the severest symptoms of affection in every other organ will be excited during its progress, is a fact known to every practitioner. Can any one, except a general physician, do justice to a disease thus complicated? But, if this question be unavoidably answered in the negative, why is the treatment of the insane erected into a separate department? Why is a principle of exclusion authorized which must be injurious to the cause of science, both in as far as insanity is concerned and in respect to general disease, on which this malady is calculated to throw lights?

"Division of labour never contributes to skilfulness where it isolates studies, which, if united, might bestow reciprocal support."

We have been much pleased with the style and method of this volume, and regret that the Doctor did not extend his sketches into regular portraits. We trust that he may have an opportunity of further investigating this disease; as, with his talents and ability, we may look forward with confidence to the elucidation of phænomena, the nature of which has hitherto been hidden in the most profound obscurity.

